# Supplementary material for: Identification of photoperiod-induced specific miRNAs in the adrenal glands of Sunite sheep (Ovis aries)
Source: Front Vet Sci. 2022 Jul 22;9:888207. doi: 10.3389/fvets.2022.888207 (PMC9354845; doi:10.3389/fvets.2022.888207)
Supplement: Supplementary file 1 [file Data_Sheet_1.ZIP › Supplementary file/miRDeep2 file.zip!/miRDeep2 file/Know_Structure/SPLP42A3/oar-mir-218a.pdf]

miRBase precursor	: oar-mir-218a
Total read count	: 71899
oar-miR-218a read count	: 71898
remaining reads	: 1

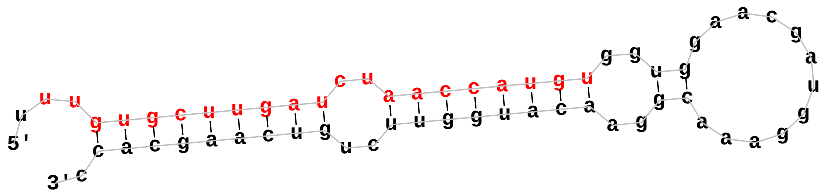

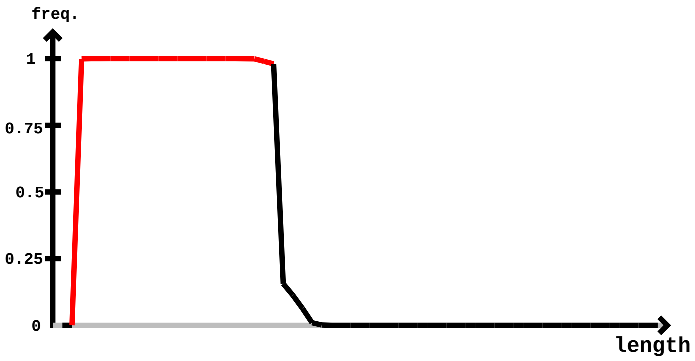

oar-miR-218a

```
5'- uuugugcuugaucuaaccaugugguggaacgauggaaacggaacaugguucugucaagcacc   -3'    exp
    ...(((((((((..((((((((..((............))..))))))))..)))))))))).    reads      mm         sample
    uuugugcuugaucuaaccaugu........................................    8          0          seq
    Guugugcuugaucuaaccaugu........................................    1          1          seq
    .uugugcuugaucuaacA............................................    6          1          seq
    .uugugcuugaucuaacc............................................    22         0          seq
    .uugugcuuUaucuaacc............................................    1          1          seq
    .uugugcuugaucuaacca...........................................    30         0          seq
    .uugugcuugaucCaacca...........................................    1          1          seq
    .uugugcuugaucuaacAau..........................................    4          1          seq
    .uugugcuugaucuaaccau..........................................    661        0          seq
    .uugugcuugaucuaacAaug.........................................    2          1          seq
    .uugugcuugaucuaaccaug.........................................    691        0          seq
    .uugugcuugaucuaaccaugu........................................    59060      0          seq
    .uugugcuugaucuaacAaugu........................................    172        1          seq
    .uugugcuugaucuaaccaugug.......................................    3112       0          seq
    .uugugcuugaucuaaccaugugg......................................    3505       0          seq
    .uugugcuugaucuaaccauguggu.....................................    3859       0          seq
    .uugugcuugaucuaaccaugugguU....................................    579        1          seq
    .uugugcuugaucuaaccauguggug....................................    20         0          seq
    .uugugcuugaucuaaccaugugguUg...................................    91         1          seq
    ..ugugcuugaucuaaccaugu........................................    13         0          seq
    ..ugugcuugaucuaaccaugugg......................................    27         0          seq
    ..ugugcuugaucuaaccauguggu.....................................    16         0          seq
    ..ugugcuugaucuaaccaugugguU....................................    9          1          seq
    ..ugugcuugaucuaaccauguggug....................................    1          0          seq
    ..ugugcuugaucuaaccaugugguUg...................................    1          1          seq
    ...gugcuugaucuaaccaugu........................................    2          0          seq
    ...gugcuugaucuaaccaugugguU....................................    1          1          seq
    ....ugcuugaucuaaccaugu........................................    1          0          seq
    ....ugcuugaucuaaccaugugg......................................    1          0          seq
    ....ugcuugaucuaaccaugugguU....................................    1          1          seq
    ......................gguggaacgauggaaacggaac..................    1          0          seq
```
